# Psychophysiological mechanisms underlying the failure to speak: a comparison between children with selective mutism and social anxiety disorder on autonomic arousal

**DOI:** 10.1186/s13034-021-00430-1

**Published:** 2021-12-28

**Authors:** Felix Vogel, Christina Schwenck

**Affiliations:** Department of Special Needs Educational and Clinical Child and Adolescent Psychology, Justus-Liebig-University Giessen, Otto-Behaghel-Straße 10, 35394 Giessen, Germany

**Keywords:** Selective mutism, Social anxiety disorder, Social phobia, Social stress, Cognitive variables, Physiological response

## Abstract

**Background:**

Selective mutism (SM) has been conceptualized as an extreme variant of social anxiety disorder (SAD), in which the failure to speak functions as an avoidance mechanism leading to a reduction of intense fear arousal. However, psychophysiological studies in children with SM are scarce and physiological mechanisms underlying the failure to speak are largely unknown. In contrast, children with SAD are characterized by a combination of a chronically elevated physiological arousal and a blunted physiological fear response to social stress. Due to the large overlap between SM and SAD, similar mechanisms might apply to both disorders, while differences might explain why children with SM fail to speak. The aim of our study is to investigate psychophysiological mechanisms of the failure to speak in children with SM.

**Methods:**

We assessed in a total of N = 96 children [8–12 years, SM: n = 31, SAD: n = 32, typical development (TD): n = 33] resting baseline arousal in absence of social threat and the course of physiological fear response in two social stress paradigms, differing in terms of whether the children are expected to speak (verbal task) or not (nonverbal task).

**Results:**

Children with SM were characterized by increased tonic arousal compared to the other two groups, and by a more inflexible stress response in the nonverbal but not in the verbal task compared to TD-children. Further analyses revealed that children with SM who did not speak during the verbal task already demonstrated reduced arousal in anticipation of the verbal task.

**Conclusion:**

The increased tonic arousal generalized to non-social situations in SM could indicate a long-term alteration of the autonomic nervous system. Furthermore, the differential physiological stress response may indicate that silence acts as a maladaptive compensatory mechanism reducing stress in verbal social situations, which does not function in nonverbal situations. Our findings support the idea that the failure to speak might function as an avoidance mechanism, which is already active in anticipation of a verbal situation. Treatment of SM should take into account that children with SM may suffer from chronically elevated stress levels and that different mechanisms might operate in verbal and nonverbal social situations.

**Supplementary Information:**

The online version contains supplementary material available at 10.1186/s13034-021-00430-1.

## Introduction

Selective mutism (SM) is a mental health disorder in which affected children fail to speak in certain social situations where they are expected to, but their speech remains unaffected in other situations [1]. The disorder is associated with a chronic course of increased psychopathological symptoms persisting into adulthood and resulting in impairments in academic and socio-emotional development [2–4]. Previous research suggests a prevalence of approximately 1% [5], although the occurrence of SM is probably underestimated [6]. With the introduction of the DSM-5 [1], SM was classified among anxiety disorders for the first time. The reason for this was evidence that SM shares numerous similarities with other anxiety disorders, particularly social anxiety disorder (SAD) [5]. SAD is characterized by a marked fear of being evaluated by others in social situations as well as physiological symptoms [1]. The overlap between SM and SAD is, for example, reflected in high comorbidity rates up to 100% of SAD in children with SM [7] and findings that social fear is a central phenomenon in children with SM [8]. Additionally, etiological similarities between both disorders exist such as the increased risk of the temperament of Behavioral Inhibition (BI) during early childhood [9].

In light of this extreme overlap, hitherto an unresolved question is why children with SM fail to speak in certain social situations, whereas children with SAD do not, despite both clinical groups experiencing marked social fear. In this context, SM has been conceptualized as an extreme variant of SAD with an extreme fear and overarousal in social situations, in which failure to speak functions as an avoidance mechanism leading to a reduction in fear arousal [10–15]. While the assumption of SM as an extreme version was based on findings that children with SM are rated by clinicians as more anxious in social situations than children with SAD [11, 14], comparable levels of self-reported social trait anxiety between both groups speaks against this notion [11, 14, 16]. However, recent studies on children with SM suggest that it is important to differentiate between social situations in which children are expected to speak (verbal) and situations which do not require speech (nonverbal) [10, 13, 17]. During nonverbal social situations, children with SM and SAD report comparable levels of state anxiety [13] and are rated as comparably anxious by parents [17], teachers [17] and clinicians [10], but still show increased levels of fear compared to children with typical development (TD) [10, 13, 17]. In verbal social situations, children with SM are rated as more anxious by clinicians [10] and report higher levels of fear than children with SAD [13]. Therefore, research supports the assumption that children with SM show an extreme level of fear compared to children with SAD, even if this only applies to verbal situations. This suggests that failure to speak is related to extreme fear in verbal situations, which implies that it is part of a dysfunctional fear response. Consequently, differences in fear responses between SM and SAD may provide insights into mechanisms of failure to speak. However, support mainly comes from studies using data based on self-report or behavioral observations, and few quasi-experimental studies exist, which use objective measures of fear such as physiological processes. When faced with a fear-inducing situation, a fear response occurs in order to cope with the threat. Immediate fear responses to threat in the form of fight, flight or freeze are mediated by the autonomic nervous system [18], which consists of a sympathetic and a parasympathetic part. The presence of threat leads to the activation of the autonomic nervous system, which results in an increase in sympathetic and decrease of parasympathetic activity [18]. This change of arousal in response to a fear-related situation already initiates in anticipation of the threat [19, 20] and is called reactivity (in experimental designs defined as change from baseline to confrontation with threat). Reactivity is associated with a higher sympathetic activity indicated by an increase in skin conductance level (SCL) and heart rate (HR) and a reduced parasympathetic activity indicated by a decrease of the respiratory sinus arrhythmia (RSA) [18, 21]. Recovery from threat leads to decrease of SCL and HR and increase of RSA. Aside from the phasic change in physiological arousal in a fear-inducing situation (reactivity or recovery), the basic level of arousal in the absence of stress is called tonic autonomic arousal [22]. Disorder-specific models of other anxiety disorders [23], as well as psychophysiological theories of anxiety in general, propose physiological mechanisms to be involved in the symptomatology of anxious individuals [22, 24, 25]. In contrast, no empirically based model exists in SM and mechanisms of failure to speak are largely unclear, as psychophysiological research in children with SM is scarce. The assumption that failure to speak occurs due to more intense fear and associated overarousal in children with SM compared to children with SAD implies that children with SM have *higher autonomic reactivity* during an expectation to speak. At the same time, disorder-specific models of SAD suggest that individuals with SAD exhibit increased reactivity compared to non-anxious individuals [23]. However, the few existing studies on autonomic activity in SM [14, 26] do not support the notion of a higher reactivity in SM compared to SAD as a mechanisms of failure to speak. In this context, Heilman et al. [26], who did not include a group of children with SAD, found no difference in reactivity during a non-standardized verbal social stress task between children with SM and typical development (TD). Young et al. [14] did not examine the change of autonomic arousal from the baseline to a non-standardized speech demanding task, but only investigated the level of arousal during both phases. Therefore, no conclusions about reactivity of the autonomic arousal can be drawn from this study. Also against the assumption of SAD models, most psychophysiological studies do not show a higher reactivity in children with SAD compared to children with typical development (TD) during social situations [17, 18, 21–24].

In contrast to the higher reactivity suggested by disorder-specific theoretical assumptions, psychophysiological theories of anxiety propose a *lower reactivity* to be indicative of a pathological fear response [18, 22]. In line with the idea that a fear response is an adaptation to the fear-inducing situation, it is assumed that strong and flexible reactivity is functional, as long as the arousal recovers quickly after the situation. Instead, they propose the combination of a *tonic elevated autonomic arousal* (in the absence of stress) and a blunted response of the autonomic nervous system to stress, consisting of a *lower reactivity* (lower increase of arousal) and *slower recovery* to be indicative of pathological anxiety [18, 22]. Tonically increased autonomic arousal is associated with reduced responsiveness of the autonomic nervous system [22]. The combination of high tonic arousal and blunted response is also known as restrictive autonomic flexibility, which can transdiagnostically be found in anxious individuals and has been interpreted as chronic dysregulation of the autonomic nervous system [27, 28]. While autonomic flexibility has not directly been studied in children with SM, research in children with SAD consistently indicates a restricted autonomic flexibility during social stress. Both an increased tonic arousal [27, 28] as well as a blunted response to social stress, with lower physiological reactivity and a subsequent slow reduction of arousal can be found in children with SAD [28–30], while the restriction of autonomic flexibility is positively related to the level of state anxiety [31]. However, physiological studies in socially anxious children have been criticized for being limited in indicating whether arousal is actually tonically increased or whether arousal is already influenced by the unfamiliar laboratory setting (e.g., presence of strangers) that induces stress in this clinical group [32]. For this reason, Asbrand et al. [32] examined resting arousal in a familiar environment and the absence of social stress, and found that children with SAD showed higher autonomic resting arousal here as well. This tonically increased arousal, even in non-stress inducing situations, can already be observed in toddlers with a high level of the temperamental BI feature [33–35], which is a precursor for both SM and SAD.

Given that inflexibility of the fear response, rather than high reactivity, is indicative of pathological anxiety, and that children with SM experience an extreme fear in verbal social situations compared with SAD, SM might be associated with an even stronger inflexibility of the fear response. However, psychophysiological studies focusing on autonomic activity in children with SM have not yet examined tonic autonomic arousal in the absence of stress, nor do they include direct analyses of both reactivity and recovery over the course of a stressful situation [14, 26]. Young et al. [14] found no significant differences in level of autonomic arousal between children with SM, SAD, and TD during a baseline in a laboratory setting, whereas statistical power was limited due to the very small sample size. The authors did not examine arousal after the verbal stress task and did not analyze differences between phases, so no conclusions about reactivity or recovery can be drawn from this study. Heilman et al. [26] examined baseline arousal in a laboratory setting in combination with physiological responses during and after a verbal social stress task. Although the authors examined reactivity to the verbal stress task, they did not examine recovery. The authors found a higher HR as well as a lower RSA in children with SM compared to children with TD during a baseline condition. Children with SM showed lower RSA after the task, indirectly suggesting a slower recovery from the stressor. However, the authors did not correct for differences in level of arousal between groups (baseline correction), did not use a standardized social stress task and did not control for speech production, which is known to influence physiological responses [36]. Taken together, these findings also seem to indicate a restrictive autonomic flexibility in children with SM, although not all components in SM have yet been investigated. Furthermore, baseline measurements of both studies were performed at an unfamiliar place and in the presence of strangers, both of which induce symptoms in children with SM. In this respect, it is questionable whether the findings of Heilman et al. [26] indicate a chronically increased arousal in children with SM or are a consequence of the factors inducing symptoms in SM. Complementary to the assumption that SM is associated with extreme fear, it has been proposed that failure to speak is an avoidance mechanism [5, 12, 14, 37]. Here, the assumption is that the intense fear experienced by children with SM during a verbal social situation is reduced by the failure to speak [14]. While this is supported by evidence from a qualitative study in which individuals with SM report in some cases that failure to speak is associated with the experience of safety [8], there is also initial evidence from the above-mentioned psychophysiological studies. Children with SM showed a comparable level of autonomic arousal as children with TD during verbal tasks of both above-mentioned physiological studies and a lower arousal than children with SAD in the study of Young et al. [14]. The lower arousal compared to children with SAD has especially been interpreted in favor of considering failure to speak as avoidance mechanism. However, the design of both studies [14, 26] is not completely suitable for testing this assumption due to two reasons: (1) they did not examine a detailed course of the fear response. While an increase in autonomic arousal already starts in anticipation of a fear-inducing situation, it is implicit in the assumption of the avoidance mechanism that arousal decreases again as soon as the expectation to speak sets in and the child fails to speak. Therefore, it is important to capture the course of anticipation of the fear-inducing situation and the situation in which the child does not speak. (2) Both studies did not control for all important confounding variables. In this context, it is especially important to control speech production during the verbal stress task, as it has an impact on the physiological response. Additionally, in order to investigate a possible avoidance mechanism, it would be important to consider a nonverbal social stress task, which also induces fear in children with SM but cannot be avoided by failure to speak. Given that previous studies have not done this, a comparison of fear responses between these situations could provide insight into whether reduction happens only in the verbal situation that can be avoided by failure to speak.

Therefore, the current study aims to examine the responses of the autonomic nervous systems of children with SM compared to children with SAD and TD. In addition to tonic autonomic arousal during rest, the fear response (reactivity and recovery) is investigated with regard to both verbal and non-verbal social standardized stress situations. Additional to autonomic flexibility, we examine the course of the fear response comparing both types of situations in order to capture a potential reduction of arousal through failure to speak in SM. In order to gain detailed insight into the course of the fear response, we analyze arousal in different phases of the fear-inducing situations (baseline, anticipation, performance, recovery). While a story is to be retold in the verbal task, the nonverbal task consists of drawing a previously heard story, so that it cannot be avoided by failure to speak. To induce social stress, both tasks take place in front of an unfamiliar experimenter and children had been told that other children will later evaluate the recorded tasks. Furthermore, we analyze and control for possible baseline differences as well as confounding variables such as age, gender, and the number of spoken words for the analysis of physiological response. Furthermore, we take into account the level of BI retrospectively assessed for toddlerhood, as this has to be shown to have an influence on the tonic arousal level.

We aim to address the following hypotheses:

1. Autonomic flexibility: We aim to shed light on the fear response according to the concept of restricted autonomic flexibility in children with SM:
  1.1. Resting Arousal:
    - We expect children with SM and SAD will show higher tonic autonomic arousal (indicated by elevated HR and SCL and reduced RSA) during a rest period in a non-threatening environment compared to TD children.
    - To investigate whether the higher arousal is rooted in the temperament BI, which is a precursor to both disorders, we test whether the level of BI can predict arousal beyond the symptomatology of SM and SAD.
  1.2. Reactivity and recovery during nonverbal social stress: We expect that children in the clinical groups will show a lower reactivity and a slower recovery on sympathetic and parasympathetic markers during nonverbal social stress compared to TD children (SM = SAD > TD).
  1.3. Reactivity and recovery during verbal situations:
    We further expect the children with SM will show a lower reactivity and slower recovery during verbal stress compared to both children with SAD and children with TD and we expect children with SAD will show lower reactivity and slower recovery compared to children with TD (SM > SAD > TD).
2. Avoidance: We aim to investigate the failure to speak as a potential disorder-specific mechanism in SM. In order to address limitations of previous physiological studies in SM [14, 26], we aim to investigate the course of the fear response in children with SM and compare it between a verbal and a nonverbal social stress situation. Hereby, we want to identify whether a reduction of the physiological arousal occurs with the onset of an expectation to speak after it increases in anticipation of the fear-inducing situation.
  2.1. Reduction of arousal: We hypothesize that autonomic arousal in children with SM will increase (increase of HR and SCL and decrease of RSA) in anticipation of the speech demanding situation and will decrease (decrease of HR and SCL and increase of RSA) during the speech demand (performance of verbal task).
  2.2. Comparison of paradigms: We hypothesize that a reduction from anticipation to performance of the stress task in children with SM will only occur in a verbal task with the expectation to speak, but not in a nonverbal social stress task that cannot be avoided by failure to speak. We therefore assume that the difference in autonomic arousal between anticipation and performance of the verbal social stress task (performance–anticipation) in children with SM will be larger than in the nonverbal situation.

## Methods

### Sampling and recruitment

Individuals were recruited throughout the state of Hessen via psychiatric clinics, psychotherapeutic outpatient clinics, schools, speech therapists, advertisements, mailings to households, and from an existing database. Compensation in the form of a €20 voucher was offered for participation in the main part of the study. The local ethics committee of the University of Giessen approved the study. All interested families were provided with a link with information about the study procedure and invited to participate in a brief online questionnaire in order to screen for symptoms of SM (using the Frankfurt Scale of Selective Mutism) and SAD (based on the DSM-5 criteria). Potential mental health disorders were diagnosed later in the study based on a comprehensive clinical interview (Diagnostic Interview for Mental Disorders in Children and Adolescents; Kinder-DIPS) conducted with the parents. BI was assessed through parent-report based on the Retrospective Infant Behavioral Inhibition Scale (RIBI) and social anxiety through self-report based on the Social Phobia and Fear Inventory for Children (SPAI-C). Informed consent was obtained from all caregivers both before the brief online questionnaire and before the main part of the study. From n = 159 caregivers of children between 8 and 12 years who participated in the online questionnaire, a total of n = 106 families agreed to participate in the main part of the study, during which the social stress tasks and a clinical interview for the assessment of diagnoses were conducted. This appointment took place at the families’ homes, so as to ensure the acquisition of a resting measurement in a non-threatening environment [32], and to facilitate participation due to the familiarity of the environment. Due to technical difficulties in the physiological measurement, complete physiological data of n = 6 subjects were not available and were therefore excluded from the present study. In addition, n = 4 individuals were excluded, as they displayed other mental illnesses than SM or SAD during further clinical assessment and therefore could not be assigned to the TD group or either of the two clinical groups. The final sample consisted of N = 96 individuals (age: M = 9.48, SD = 1.28), of whom n = 31 were assigned to the SM group, n = 32 to the SAD group and n = 33 to the TD group, based on the conducted clinical interview. Of the n = 31 subjects with SM, n = 24 (80%) also fulfilled the diagnosis of SAD, whereas the precondition for assignment to the SAD group was the absence of comorbid SM. None of the children with SAD had a lifetime diagnosis of SM. The children of the TD group did not fulfill the criteria for any mental health disorder. It is important to emphasize that there is a risk to overdiagnose SAD in children with SM, as clinicians tend to interpret the failure to speak of children with SM as anxiety [16]. Given that the clinical interview used in the study at hand considers both verbal and nonverbal social situations for diagnosing SAD, we might have reduced the risk of overdiagnosing SAD in the SM group. An overview of the comorbidities is included in Additional file 1. Groups did not differ with respect to age and gender and clinical groups did not differ regarding the number of comorbidities. Table 1 shows an overview of the sample characteristics.

**Table 1:** Sample characteristics

	SM	SAD	TD	*P*	Post-hoc
n	31	32	33		
Age	9.07 (1.15)	9.50 (1.19)	9.79 (1.41)	0.075	–
Gender (f/m)	19/12	18/14	18/15	0.889	–
FSSM-DS	7.71 (1.66)	4.44 (2.59)	0.70 (1.21)	< 0.000	SM > SAD > TD
FSSM-SS (z-score)	0.63 (.89)	0.23 (0.70)	− 0.81 (0.28)	< 0.000	SM > SAD > TD
SPAI-C sum score	18.86 (9.42)	18.92 (10.12)	9.24 (5.97)	< 0.000	SM = SAD > TD
RIBI sum score	39.06 (13.59)	41.91 (15.99)	23.84 (12.86)	< 0.000	SM = SAD > TD
Number of words	93.09 (86.43)	155.00 (65.46)	217.76 (81.55)	< 0.000	SM < SAD < TD

*FSSM-DS* Frankfurt Scale of Selective Mutism-Diagnostic Scale, *FSSM-SS* Frankfurt Scale of Selective Mutism-Severity Scale, *SPAI-C* social phobia and anxiety inventory for children, *RIBI* Retrospective Infant Behavioral Inhibition Scale, *SM* selective mutism, *SAD* social anxiety disorder, *TD* typical development

### Procedure

**Fig. 1:**
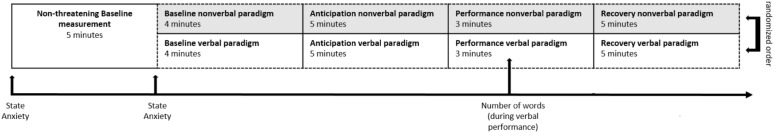
Procedure of social stress paradigm and measured constructs

After completing the online questionnaire, the families were visited in their homes by two experimenters. At the beginning of the appointment, parents and children were again informed that the aim of the study was to investigate bodily reactions of stress in children with selective mutism in different situations. Both were informed that the participating child would perform various tasks, either simply lying down, drawing something, or retelling a story, and that meanwhile, body responses would be measured. After clarification of the study contents and the confirmation of the informed consent of parents and children, electrodes were applied to the children in order to conduct the physiological measurements (see section below). Then, a measurement was performed in a resting state. For this, the individuals were instructed to lie down on a mat for 5 min without talking to the caregiver present in the room. During the resting measurement, the experimenters were not present in order to reduce possible stress caused by the presence of strangers. Before and after the resting measurement, the children were asked to rate their anxiety level on an anxiety thermometer and to assess the extent of their arousal using an analog scale adapted from the arousal dimension of the self-assessment manikin. Both the anxiety thermometer and the self-assessment manikin analog scale are well-established scales used in research to assess state anxiety and arousal in children with anxiety disorders [38, 39]. Subsequently, one experimenter together with the caregiver went to a separate room, where a structured clinical interview (Kinder-DIPS) was performed with the caregiver. The experimenters who conducted the interview were graduate students of psychology who were trained and supervised by a clinical psychologist. Meanwhile the other experimenter performed a social stress paradigm with the child alone (without a caregiver present). The child went through a standardized experimental protocol (see Fig. 1) consisting of a verbal and a non-verbal social stress paradigm, with the order being randomized (see “Materials” section). Importantly, the participating children did not know whether the experimenter was aware of their difficulties in speaking, so the children may have been less influenced by a possible expectation of the experimenter. Physiological data were continuously recorded during the stress paradigm. During the verbal task, the number of spoken words was counted. After both performance phases the children completed a short questionnaire on attentional focus, which is not relevant for the aim of the present study. The paradigm was followed by an eye-tracking paradigm and assessment of cognitive variables, which are published elsewhere.

### Materials

#### Social stress paradigm

The stress paradigm of the current study was adapted from the Trier Social Stress Test for Children (TSST-C), which has been proven to be highly effective for stress induction [40] and is most commonly used in psychophysiological research in socially anxious children [28–30]. We had to adapt this paradigm because for our hypotheses, a differentiation between a verbal and nonverbal stress task is required and no validated procedure exists for this yet. Differences between the paradigm of the current study and the TSST-C as well as the rational behind each adaptation are displayed in Additional file 1: Table S5. For the construction of our paradigm, we created a standardized protocol (see Fig. 1) and considered stress-inducing elements of the TSST-C such as the use of a video recorder or a cover story with standardized phrases to elicit social stress (see description below). Children were seated at a table opposite the experimenter and also remained seated for the entire duration of both stress paradigms, which were conducted consecutively without any time break. Both the verbal and non-verbal paradigms were intended to be as comparable as possible and began with a baseline phase (4 min) in which relaxing nature videos were shown on a screen. Subsequently, the children were informed that they now had time to prepare (anticipation: 5 min) for retelling and continuing a story presented to them in picture form (verbal) or painting a story read to them (non-verbal). Furthermore, the children were told that they were supposed to complete the task in a few minutes, each time in front of the experimenter and a video camera. A cover story was used for both paradigms, suggesting that the children would be recorded on video and that other children of the same age would see the video later and evaluate it. After the preparation time, the experimenter turned on the video camera and instructed the child to start telling the story or paint the picture story (performance: 3 min). For this purpose, TSST-C standardized phrases such as “other children will evaluate you afterward, so make an effort” were used to induce social-evaluative stress. During the verbal paradigm, if the child had not spoken after 20 s or had finished telling the story before the time was up, the child was notified in a standardized manner that “there is still time left”. During the recovery phase (5 min), the child again watched relaxing nature videos. Relaxing nature videos during both the baseline and recovery phases were introduced with the instruction that children could relax while watching the videos. The baseline phase of the second paradigm followed directly after the recovery phase of the first paradigm had ended.

#### Psychometric measures

The *Diagnostic Interview for Mental Disorders in Children and Adolescents* (Kinder-DIPS) [41, 42] is a structured clinical interview used to diagnose mental health disorders in children and adolescents. An evaluation is possible according to both DSM-5 and ICD-10. The interview has a good to very good inter-rater reliability [43] and has a high acceptance by both interviewers and respondents [43, 44]. In the present study, the Kinder-DIPS was used to diagnose mental health disorders or to rule out the presence of such a disorder. Individuals who met the DSM-5 criteria for SM were assigned to the SM group, regardless of whether they also met the criteria for SAD. Individuals who met only the DSM-5 criteria for SAD but not for SM were assigned to the SAD group, and individuals who did not meet the criteria for any mental health disorder were assigned to the TD group.

The *Frankfurt Scale of Selective Mutism* (FSSM) [45] is a questionnaire for assessing the symptoms of SM in children and adolescents aged 3–18 years, based on a parental judgment. The FSSM comprises a diagnostic scale (DS) consisting of ten dichotomous items (yes/no) related to the child's general speech pattern and is available in three developmentally adapted versions (kindergarten children from 3 to 7 years old, school children from 6 to 11 years old and individuals from 12 to 18 years old). The versions used in the present study for children between 6 and 11 years of age and adolescents between 12 and 18 years of age have a cut-off value of 7 and 6 respectively, indicating the presence of SM. Additionally, the FSSM includes a severity scale (SS) that can be used to assess the symptom severity of SM. This consists of either 41 or 42 questions on speaking behavior in various situations, depending on the developmentally adapted version. Questions are answered on a 5-point Likert scale and a total sum score can be calculated. In the present study, we formed z-scores of SS to integrate the sum scores of the different developmentally adapted versions into a common total score. The ROC analysis performed by the authors confirms a very good differentiation between children with SM (M = 8.2, SD = 1.5), SAD (M = 3.6, SD = 2.5) and children without a mental health disorder (M = 0.5, SD = 0.8) and shows excellent reliability (Cronbach’s α = 0.90–0.98.). For our sample, the reliability for the FSSM was also excellent (α = 0.951–0.959).

We used the German version of the *Social phobia and fear inventory for children (SPAI-C)* [46, 47], which measures symptom levels of social anxiety via self-report. The questionnaire consists of 26 items with a 3-point Likert scale on different social situations and an overall score of 52 can be reached. Both reliability (Cronbach’s α = 0.92) and validity are considered high [47–49]. Beidel et al. [46] reported a cut-off score of 18, which is able to differentiate between children with SAD and non-socially anxious children. For our sample, reliability was also excellent (α = 0.959).

The *Retrospective Infant Behavioral Inhibition Scale (RIBI)* [45, 50] is a questionnaire based on a retrospective parental report to assess the child's behavioral inhibition (BI) for the first two years of life. The RIBI has a 3-factor structure and includes the subscales Distress to Novelty, Fear, and Shyness in addition to the total score scale for the expression of BI. Parents answer 20 items on a 5-point scale (0 = Yes, 1 = more likely Yes, 2 = partly, 3 = more likely Not, 4 = Not). The test has excellent reliability (> 0.90) and convergent validity in the form of positive correlations with questionnaires as well as laboratory tests for BI at 14 months of age [50]. In our sample, the reliability was also excellent (α = 0.909).

#### Psychophysiological measures

All physiological measures were recorded with an acquisition sample rate of 1000 Hz simultaneously using the BIOPAC system MP160 and the portable bionomadix modules for ECG, respiration, and electrodermal activity. A 3-point ECG and two 2Ag/Cl electrodes for measurement of electrodermal activity were installed on the children’s thoraxes and index and middle fingers of the non-dominant hands. For the calculation of RSA, respiration was recorded using a respiratory belt on the child’s chest. The physiological data were pre-processed using the Acqknowledge software (BIOPAC). All data sets were visually examined for artifacts, missing or obsolete QRS complexes were corrected and HR was calculated in beats per minute (BPM) based on an ECG channel. The electrodermal activity data was adjusted in Acqknowledge via linear regression for the increasing trend of SCL. The RSA was calculated using multi-epoch spectral analysis.

### Data analysis

#### Data reduction and statistical analysis

All statistical analyses were performed in SPSS 26.0. We compared groups on age, symptom scores (FSSM, SPAI-C), temperament (RIBI), and the number of spoken words using univariate ANOVA and Bonferroni-corrected post-hoc tests. Number of words were counted from a tape recording, which was conducted during the performance phase of the verbal task. The reason for considering the number of words is that speech production itself can have an impact on autonomic processes [36]. Gender distribution was compared based on a chi-square test.

From the pre-processed physiological data, mean values for HR, SCL and RSA were calculated for the individual phases of both paradigms (verbal: BL_verb_, Ant_verb_, Perf_verb_ Rec_verb_; nonverbal: BL_nonverb_ Ant_nonverb_ Perf_nonverb_ Rec_nonverb_) and the initial resting phase. Outliers (z-value ± 3 SD) were excluded for the physiological variables, according to previous research [29]. For HR, *n* = 0 cases were excluded; for SCL *n* = 4 cases (SM: *n* = 2; SAD: *n* = 2); for RSA *n* = 4 cases (SM: *n* = 2; SAD: *n* = 2).

*Resting arousal (H1.1 a and b)* Initially, we checked whether the groups differed in terms of their subjective anxiety levels during the resting measurement in the absence of stress. This is important to rule out that possible differences in physiological resting arousal are not due to higher subjective anxiety. The mean values of the state-anxiety and experienced arousal before and after the resting measurement were z-standardized and integrated into an anxiety measure consisting of an emotional and bodily component and then compared between groups using independent sample *t* tests. To counteract the risk of alpha error accumulation due to multiple testing, autonomic arousal during rest was compared between groups using a multivariate analysis of variance (MANOVA) including all three measures (HR, SCL, RSA) and group (SM, SAD, TD). Significant group differences were decomposed using Bonferroni-corrected post-hoc tests. To test whether potentially increased resting arousal was due to the level of BI, we first calculated correlations between BI and resting arousal. Given that BI was not significantly correlated with any of the three measures during rest, the multiple regressions were not performed.

*Reactivity and recovery during nonverbal and verbal social stress (H1.2 & H1.3)* In order to minimize the accumulation of alpha errors by multiple testing, we largely based the statistical calculation of the physiological fear response as a function of group and phase on the established procedure in research on children with SAD [29, 30]: (1) to control baseline differences between the groups, we used a MANOVA including all measures (HR, SCL, RSA) and groups (SM, SAD, TD) to test whether autonomic arousal during baselines for the verbal and nonverbal paradigms differed between groups. Furthermore, a baseline correction was performed by subtracting the baseline means of the corresponding paradigm from the remaining phase means (verbal: Ant_verb_ − BL_verb_, Perf_verb_ − BL_verb_, Rec_verb_ − BL_verb_; nonverbal: Ant_nonverb_ − BL_nonverb_, Perf_nonverb_ − BL_nonverb_, Rec_nonverb_ − BL_nonverb_). This was done to control expected level differences between groups for phase analysis. (2) We performed a MANOVA with all physiological measures (HR, SCL, RSA) for each paradigm (verbal, nonverbal) preceding univariate analyses to keep alpha error accumulation at a minimum. The physiological measures were not combined into a common parameter, but were included as independent parameters. In both MANOVAs as well as in subsequently conducted mixed ANOVAs, we included group (SM, SAD, TD) and phase (baseline, anticipation, performance, recovery) as factors. Because we were interested in examining whether groups differ in terms of the course of their fear response (reactivity and recovery), we checked based on the MANOVAS whether there was a group × phase interaction with respect to all physiological measures per paradigm. (3) Significant effects of MANOVA on group × phase were then decomposed according to previous research [29]: in order to analyze reactivity (defined as the *change from baseline to performance*) and recovery (defined as the *change from performance to recovery*) we conducted separate mixed ANOVAs (group × phase) for reactivity (baseline, anticipation, performance) and recovery (performance, recovery) per measure and conducted Bonferroni-corrected post hoc tests for comparison of single phases between groups. MANOVAs and ANOVAs were Greenhouse–Geiser corrected. In addition, to control for possible confounding variables, we correlated reactivity (performance–baseline) and recovery (recovery–performance) of each measure with age and gender. Furthermore, we correlated the number of words with the physiological measures during the performance phase of the verbal paradigm to control for a possible influence of speech production. None of these variables correlated with the physiological measures, so we did not include them as control variables for the analyses.

*Avoidance (H2.1 & H2.2)* To examine the first hypothesis (H2.1) on failure to speak as an avoidance mechanism, we compared autonomic arousal of children with SM between phases (baseline, anticipation and performance) of the verbal stress paradigm using paired *t* tests. To check whether a possible reduction occurs only in the verbal and not in the nonverbal paradigm (H2.2), we compared the phases also in the nonverbal paradigm with paired *t* tests. Furthermore, to compare the reduction of anticipation to performance between the two paradigms (H2.2), we calculated the difference between anticipation–performance for HR, SCL and RSA for both paradigms. Because the use of score differences has been criticized and their reliability mainly depends on the reliability of the underlying measures [51], we checked the internal consistencies of all three physiological measures per phase. These ranged between α = 0.876 and 0.995, which implies that the difference scores are reliable as well [51]. We compared the differences using paired *t* tests. To adjust for alpha error inflation due to multiple group comparison regarding analyses of hypotheses 2, we performed a Bonferroni correction and used α = 0.003 as the significance level for paired *t* tests.

During the conduct of the experimental study, we noticed that a proportion of children with SM spoke (*n* = 23) during the verbal performance phase, although with significantly fewer words than children with SAD or TD. A smaller group of children with SM did not speak at all (*n* = 8). Because we aimed at investigating whether a *failure to speak* represents an avoidance mechanism, we conducted a further analysis comparing children with SM who have spoken and who have not spoken (mixed ANOVA with phase: BL_verb,_ Ant_verb,_ Perf_verb_ Rec_verb,_ and group: spoken vs. not spoken with Bonferroni-corrected post-hoc tests). To investigate possible differences in clinical characteristics between the two subgroups, we compared them in terms of the questionnaires used in the study, age, and gender using a MANOVA.

#### Power analysis and paradigm check

We conducted an a priori power analysis for mixed ANOVA prior to the study. Given effect sizes of *f* = 0.24–0.26 reported in previous studies on phase × group interaction as well as group differences between socially anxious and healthy children in physiological measures [29], we calculated a minimum size per group of n = 31 in order to detect an effect with a statistical power of 90%. This requirement was met in our study.

To test whether the (a) randomization of the sequence of paradigms worked and were (b) protected from sequence effects, we (a) used a chi-square test to test whether the three groups differed in terms of the number of individuals who started with the verbal or nonverbal paradigm and (b) used a repeated measurement MANOVA to test whether the physiological responses per phase averaged between paradigms differed between children who started with the verbal (*n* = 48) or nonverbal paradigm (*n* = 48). Since (a) the groups did not differ in the distribution of the order of the paradigms *X*^2^ (2, *N* = 96) = 0.762, *p* = 0.683) nor did the MANOVA show a group difference (*F*(1, 96) = 2.443, *p* = 0.122, η_p_^2^ = 0.028) or a group × phase interaction (*F*(5.2, 2.03) = 0.545, *p* = 0.652, η_p_^2^ = 0.006), randomization seems to have protected against possible sequence effects.

## Results

### Sample characteristics, psychometrics and confounding variables

The three groups did not differ with respect to age and gender (see Table 1). Children with SM and children with SAD showed higher scores than the TD group, consistent with previous research, on the psychometric measures of symptomatology of SAD and SM and level of BI. Children with SM showed a higher level on FSSM than children with SAD. As expected, children with SM spoke the fewest words during the verbal stress paradigm, followed by children with SAD and TD. The number of spoken words was not related to HR (*p* = 0.396), SCL (*p* = 0.374), and RSA (*p* = 0.850) during the performance phase of the verbal stress paradigm in which speech production was required. The correlations between reactivity and recovery of each physiological measure and age, gender, and the psychometric measures are presented in Additional file 1 for both paradigms. During the verbal stress paradigm, no significant correlations were found. During the nonverbal paradigm, the SM symptom score was related to a lower HR reactivity, while the extent of BI was related to the slower recovery of SCL.

### Resting arousal (H1.1 a and b)

Regarding the measurement at rest without the presence of a stranger, a significant group difference was found in the MANOVA (*F*(6, 172) = 2.889, *p* = 0.010, η_p_^2^ = 0.092). There was a significant group difference for HR (*F*(2, 2194.66) = 4.282, *p* = 0.017, η_p_^2^ = 0.090), but not for SCL (*F*(2, 59.56) = 0.733, *p* = 0.483, η_p_^2^ = 0.017) and RSA (*F*(2, 9.20) = 1.220, *p* = 0.300, η_p_^2^ = 0.027). Bonferroni adjusted post-hoc tests indicated that children with SM had a significantly higher HR at rest (SM: *M* = 88.28, *SD* = 12.71; SAD: *M* = 79.42, *SD* = 9.05; TD: *M* = 79.97, *SD* = 9.76) than children with SAD (*p* = 0.005) and children with TD (*p* = 0.008). There was no difference between children with SAD and TD (*p* = 0.826). Regarding subjective anxiety, there was no group difference before (*p* = 0.311) or after (*p* = 0.192) resting measurement, so the differences found here are unlikely to be due to group differences in actual anxiety levels. We did not find any correlation between level of BI and level of arousal during rest across all N = 96 children (HR: *p* = 0.809, SCL: *p* = 0.752, RSA: *p* = 0.183).

### Reactivity and recovery during nonverbal and verbal social stress (H1.2 & H1.3)

**Fig. 2:**
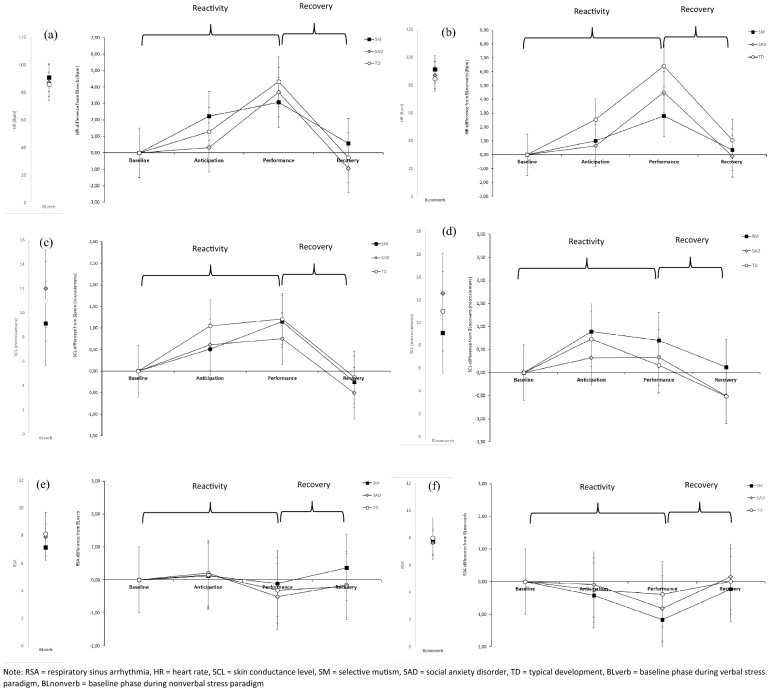
Mean baseline-values and baseline-corrected values of phases on **a** HR of verbal stress paradigm, **b** HR of nonverbal stress paradigm, **c** SCL of verbal stress paradigm, **d** SCL of nonverbal stress paradigm, **e** RSA of verbal stress paradigm and **f** RSA of nonverbal stress paradigm for SM-, SAD- and TD-group. *RSA* respiratory sinus arrhythmia, *HR* heart rate, *SCL* skin conductance level, *SM* selective mutism, *SAD* social anxiety disorder, *TD* typical development, *BL*_*verb*_ baseline phase during verbal stress paradigm, *BL*_*nonverb*_ baseline phase during nonverbal stress paradigm

(1) The MANOVA on the difference between groups in autonomic arousal during the baseline phases of the verbal and nonverbal paradigms was not significant (*F*(12, 0.225) = 1.732, *p* = 0.064, η_p_^2^ = 0.112). (2) MANOVAs on group × phase with all physiological measures showed a significant effect for phase in both the nonverbal (*F*(316.93, 1.20) = 23.414, *p* < 0.001, η_p_^2^ = 0.220) and the verbal paradigm (*F*(393.65, 2.10) = 19.669, *p* < 0.001, η_p_^2^ = 0.192), indicating that both paradigms induced a physiological fear response. For the nonverbal stress paradigm the MANOVA showed a significant group (SM, SAD, TD) × phase (baseline, anticipation, performance, recovery) interaction (*F*(67.25, 4.00) = 2.484, *p* = 0.046, η_p_^2^ = 0.056), however not for the verbal stress paradigm (*F*(40.135, 4.19) = 0.982, *p* = 0.421, η_p_^2^ = 0.024). Therefore, we decomposed the group × phase interaction using individual ANOVAS for reactivity (baseline, anticipation, performance) and recovery (performance, recovery) only for the nonverbal paradigm. For visualization, the physiological responses of the individual measures for both paradigms are shown in Fig. 2. Descriptive statistics of physiological variables of all individuals per phase are displayed in Additional file 1.

Regarding reactivity in the nonverbal stress paradigm, the individual mixed ANOVAs showed significant group × phase interactions for HR (*F*(211.35, 3.07) = 4.174, *p* = 0.007, η_p_^2^ = 0.082), but not for SCL (*F*(4.70, 3.59) = 2.291, *p* = 0.069, η_p_^2^ = 0.052) or RSA (*F*(4.91, 3.70) = 1.002, *p* = 0.405, η_p_^2^ = 0.023). Accordingly, reactivity of the nonverbal paradigm differed between groups only for HR. Bonferroni-corrected post hoc tests showed that children with SM and SAD did not differ in terms of their reactivity in the nonverbal paradigm (lower increase from baseline to performance) (*p* = 0.172). Children with SM showed significantly lower reactivity compared to children with TD (*p* = 0.023). Children with SAD did not differ in reactivity from children with TD (*p* = 0.303), but showed a lower increase from baseline to anticipation (*p* < 0.001).

The individual mixed ANOVAs for recovery did not show a significant group x phase interaction for any of the measures. Therefore, no further Bonferroni correction was necessary at this point.

### Avoidance (H2.1 & H2.2)

Based on the Bonferroni-corrected alpha level of α = 0.003, none of the measures showed a significant increase (or decrease in case of RSA) from the baseline to anticipation to speak (HR: *p* = 0.008, SCL: *p* = 0.005, RSA: *p* = 0.895). Moreover, we did not find a reduction from the anticipation of the speech demand to the performance in which speech was required (HR: *p* = 0.362, SCL: *p* = 0.005, RSA *p* = 0.857). The courses of the fear response of HR and SCL for the verbal paradigm for children with SM are displayed in Fig. 2a, c. Furthermore, for the nonverbal paradigm, there was a significant increase from baseline to anticipation for SCL (*p* < 0.001), but not for HR (*p* = 0.036) or RSA (*p* = 0.340). Consistent with our expectation, no reduction from the anticipation phase to the performance phase occurred in the nonverbal paradigm based on the Bonferroni-corrected alpha level (HR: *p* = 0.072, SCL: *p* = 0.196, RSA: *p* = 0.031). The comparisons of the difference (anticipation—performance) between the two paradigms also showed no significance, considering the corrected alpha level (HR: *p* = 0.444, SCL: *p* = 0.005, RSA: *p* = 0.051).

**Fig. 3:**
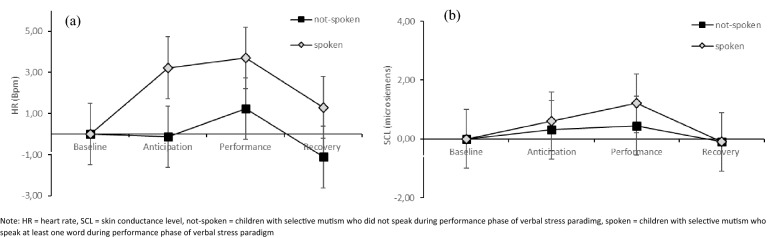
Baseline-corrected values of verbal stress paradigm for **a** HR and **b** SCL for children with SM who have spoken and who did not. *HR* heart rate, *SCL* skin conductance level, *not-spoken* children with selective mutism who did not speak during performance phase of verbal stress paradimg, *spoken* children with selective mutism who speak at least one word during performance phase of verbal stress paradigm

Regarding the further analysis of children with SM who spoke vs. children with SM who did not speak, we found a significant group difference in HR (*F*(1, 29) = 4.434, *p* = 0.044, η_p_^2^ = 0.133) between the two subgroups. We did not find an interaction between group and phase (*F*(3, 87) = 1.097, *p* = 0.355, η_p_^2^ = 0.036). A post-hoc comparison showed that children with SM who did not speak had a significant lower HR during anticipation phase (ant_verb_: *p* = 0.037; not spoken > spoken) but not during the performance phase (perf_verb_: *p* = 0.106). Regarding SCL or RSA, we did not find any group differences (SCL: *p* = 0.610; RSA: *p* = 0.817). Results are displayed in Fig. 3. To test whether this is a result specific to situations with speech demand, we also examined whether there were differences between the two groups on physiological arousal in the nonverbal paradigm. There was no difference in the nonverbal paradigm between groups for any of the measures (range of *p-*values = 0.508–0.794), indicating that children with SM who fail to speak only show reduced arousal in anticipation of a speech-demanding situation.

A post-hoc comparison between subgroups of SM on social anxiety suggests that children with SM who did not speak may be an extremely socially anxious subgroup (SPAI-C: SM-not-spoken: *M* = 25.54, *SD* = 6.86; SM-spoken: *M* = 16.32, *SD* = 9.33, SAD: *M* = 18.92, *SD* = 10.12; SM not-spoken > SM spoken: *p* = 0.014; SM not-spoken > SAD: *p* = 0.036).

## Discussion

The purpose of the present study was to investigate tonic autonomic arousal during rest as well as responses of the autonomic nervous system during a verbal and a nonverbal social stress situation in children with SM compared to children with SAD and children with TD. Hereby we aimed at gaining more insight into psychophysiological mechanisms underlying the failure to speak in SM. We expected that children with SM and children with SAD would have a restricted autonomic flexibility, consisting of a (H1.1) higher resting tonic arousal in the absence of social stress and a (H1.2) blunted response (lower reactivity and slower recovery) during social stress, compared to children with TD. We further assumed (H1.3) children with SM to show an even lower reactivity and a slower recovery during the verbal social stress task than children with SAD. Finally, we assumed that (H2.1) failure to speak in children with SM is associated with the reduction of arousal in response to a speech-demanding situation, after arousal has increased in anticipation of the situation with expectation to speak. We expected that (H2.2) the reduction of the arousal would not occur in a nonverbal situation that is unavoidable by failure to speak.

### Restricted autonomic flexibility

Our study is the first to look at all components of autonomic flexibility (tonic autonomic arousal, reactivity and recovery) during different types of social stress in children with SM. Psychophysiological theories on autonomic flexibility assume that a tonically elevated autonomic arousal and a blunted fear response are markers for psychopathology [18, 24]. The assumption that children with SM showed restricted autonomic flexibility, as has already been demonstrated for children with SAD [29] and children with other anxiety disorders [52], could be partially confirmed by our results. More specifically, children with SM showed increased tonic arousal and reduced reactivity in the nonverbal stress situation, but there was no evidence that children with SM showed slower recovery. The results on tonic autonomic arousal during rest and blunted fear response are discussed below.

#### Tonic autonomic arousal during rest

On the one hand, the presence of a restricted autonomic flexibility in children with SM is indicated by the finding of increased autonomic arousal (higher HR) during rest in the absence of social stress in children with SM compared to children with TD, which partially confirms hypothesis 1.1. Contrary to hypothesis 1.1 and findings from previous research [52, 53], there was no difference in autonomic arousal during rest between children with SAD and children with TD. Previous studies on autonomic activity in children with SM have focused primarily on social situations [10, 14], so that methodological artifacts due to situational fear-inducing factors cannot thereby be ruled out. The study at hand is the first that suggests that altered physiological processes may be generalized to non-stress-inducing situations, similar to what has already been shown for children with SAD [32]. It is important to emphasize that we took numerous precautions to minimize social stress for the children. During the measurement, no stranger but a caregiver was present and the measurement took place in a familiar environment. The minimization of stress was also reflected in finding that the subjective anxiety level did not differ between groups either before or after the measurement. Therefore, this suggests that the elevated autonomic arousal found in children with SM is not a methodological artifact induced by higher subjective fear during measurement, but actually indicates chronic dysregulated autonomic activity [52]. Tonic autonomic arousal is considered an indicator of autonomic nervous system general responsiveness, with tonically elevated autonomic arousal being associated with a more inflexible physiological response [22]. One explanation for the finding of higher tonic arousal in children with SM might be the concept of allostatic load [54] which assumes that chronic stress is associated with chronic changes in the psychophysiological balance [32, 52]. Children with SM experience increased stress in social situations and are exposed to social stress in many different places such as in public or school [15]. Similar to what Asbrand et al. [32] already assume for children with SAD, this constant and everyday experience of chronic stress could have a lasting effect on the autonomic nervous system of children with SM, resulting in increased tonic arousal. Complementing the assumption that chronic stress is associated with increased tonic arousal, there is also evidence in the literature that infants with high BI already exhibit increased tonic resting arousal [33–35]. Although we did not find a correlation between BI and tonic resting arousal across all children, level of BI and recovery of SCL were associated (see Additional file 1). This supports the idea that altered autonomic nervous system activity is already predisposed by BI temperament. Because we could only perform correlational analyses across all children due to the sample size, we cannot draw conclusion about the association between BI and physiological measures within the SM group. The extent to which physiological changes in children with SM are more likely to represent a developmental pathway linked to BI temperament, a consequence of chronic stress exposure, or a combination of both should be examined in longitudinal studies involving young children at risk for SM and SAD. Regarding the SAD group in our study, we were not able to replicate the finding of Asbrand et al. [32] that children with SAD show increased HR in a non-threatening environment. One possibility for the divergent results, though speculative, could be the rather low average severity (SAD: M = 18.92, clinical cut-off value = 18) of the children with SAD in our sample (see Table 1). Therefore, a chronically altered arousal at rest only might occur with a higher severity of the disorder.

#### Fear response (reactivity and recovery)

On the other hand, the presence of restricted autonomic flexibility in children with SM is supported by the finding of a lower reactivity of HR during the nonverbal social stress situation compared to children with TD. Since we found no difference in reactivity between children with SAD and children with TD and the groups did not differ in terms of recovery, hypothesis 1.2 is only partially confirmed. Contrary to hypothesis 1.3, we could not identify any group differences on the verbal stress paradigm and thus no more restricted autonomic flexibility in children with SM in response to a speech demand. The finding that a reduced reactivity in children with SM compared to children with TD occurs in nonverbal situations can be considered consistent with findings indicating higher levels of fear in children compared to TD children even in nonverbal situations [10, 13, 17]. The increased level of fear, which in these studies was primarily measured by questionnaires or by behavioral observation, also seems to manifest in restricted autonomic flexibility. In this context, interestingly, Milic et al. [17] showed that children with SM in a nonverbal behavioral observation task, in which no speech but gestures were expected, had a significantly longer latency to initiate behavior compared to children with TD and children with SAD. According to psychophysiological theories, restricted autonomic flexibility is associated with reduced and slowed adaptation to the threatening situation [18, 22], implying that reduced autonomic flexibility in children with SM is associated with inhibited behavioral coping with the stressful situation. The inhibition of initiation of behavior found by Milic et al. [17] in behavioral observations of children with SM, which might also be in line with findings that BI is a central phenomenon in children with SM [9], could thus be rooted in an inflexible response of the autonomic nervous system. However, it remains unclear why we did not find restricted autonomic flexibility in children with SM in verbal situations. This finding is inconsistent with evidence that children with SM experience an elevated anxiety level compared to children with SAD and TD during verbal situations [13]. Based on the finding that restricted autonomic flexibility is associated with the level of anxiety [31], we assumed that an inflexible fear response should occur especially while children with SM are expected to speak. However, these findings might be explained by a possible function of failure to speak. In this context, the literature suggests that a failure to speak may function as a maladaptive emotion regulation [37] or an avoidance mechanism that is associated with a reduction of anxiety in speech-demanding social situations [14]. Given that the level of subjective state anxiety is related to the inflexibility of the fear response, one could speculate that children with SM have regulated their anxiety level and thus their inflexible fear response in the verbal situation, albeit not deliberately, by failing to speak. Since the failure to speak is not available as a regulatory mechanism in nonverbal situations, no regulation of anxiety takes place. Consequently, a more inflexible stress response occurs that cannot be compensated for. This is also consistent with the only study in children with SM that investigated reactivity during a verbal stress task and did not find a difference between children with SM and TD [26]. Contrary to our assumptions in hypotheses 1.2 and 1.3, we did not find slowed recovery in children with SM or children with SAD compared to children with TD for either stress paradigm. In this respect, it remains an unresolved question whether all components of restricted autonomic reactivity, including reduced recovery from stress, can also be found in children with SM. Here, it is important to emphasize that the recovery phase in our study was significantly shorter than in previous studies of children with SAD [28–30], which have found a slower recovery for this clinical group. Due to the shorter recovery phase, we may not have detected possible differences between groups regarding the recovery of more slowly changing autonomic processes (e.g. SCL). Therefore, in future physiological studies in children with SM, it would be important to include recovery phases of approximately 30 min in the paradigm, as has been the case in previous studies on children with SAD, which found differences in recovery of autonomic activity [29]. Furthermore and contrary to our hypotheses, we were unable to replicate the results found in previous studies that children with SAD show a blunted autonomic response to social stress. Here, methodological differences to previous studies are also a possible explanation. In particular, this could be due to the fact that we were not able to use the validated TSST-C, which is conducted in a non-familiar environment and with multiple adults as experimenters, which in consequence is likely to lead to a stronger anxiety induction. Although both paradigms in our study also induced stress and resulted in a physiological response in all three groups, our stressor may not have been strong enough to elicit a pathological stress response in children with SAD. In consequence, although speculative, this could mean that children with SM are more sensitive to social stress and already show a dysfunctional physiological response to even a small stressor. Future research may examine this using the validated TSST-C.

#### Avoidance

Contrary to our assumption, we did not find a reduction in the arousal of children with SM when confronted with the speech demand, after arousal initially increases in anticipation of the verbal situation. Moreover, the course of arousal did not differ between the verbal and the nonverbal social stress task in children with SM, so we had to reject both hypotheses on failure to speak as an avoidance mechanism (2.1 and 2.2.). However, it is important to emphasize that not all children with SM in our sample were completely mute during the verbal stress task. This limits the analyses regarding hypotheses 2.1 and 2.2, in which we aimed at investigating whether the *failure to speak* is associated with a reduction in the arousal and thus represents an avoidance mechanism. However, the further analysis we performed to check whether the course of the autonomic fear response differs between children with SM who spoke during the verbal stress task and those who did not speak supports the assumption of the avoidance mechanism. Here, for children with SM who did not speak, the reduction in autonomic arousal, as expected in hypothesis 2.1 for all children with SM, was also not shown at the beginning of the speech demand (during the performance phase). Instead, children with SM who did not speak already had lower arousal during the anticipation phase (see Fig. 3). During the anticipation phase, the children already knew that they would soon have to speak, but no speech was yet required. This suggests failure to speak, at least in a subgroup of children with SM, functions as an avoidance mechanism, which appears in anticipation of the actual speech demand and, following this logic, is accompanied by reduced fear of expectation. Thus, the results may support the assumption in the literature that failure to speak is an avoidance mechanism [11, 14] that reduces fear effectively in the short term but is maladaptive and maintains the disorder in the long term. Alternatively, this finding could be explained by another mechanism, described in the literature as *freezing*. Freezing is a fear response that is characterized by a pattern of different physiological responses such as motor inhibition, including voice production and a decrease in heart rate [55, 56]. In our study, children with SM who failed to speak in the situation had a lower heart rate but no lower skin conductance than the SM group who spoke, which would be consistent with the physiological freezing response [57]. Furthermore, this assumption would be consistent with the descriptions in the clinical literature of some children with SM being “frozen with fear” [58]. In addition, recent research shows that a freezing reaction can occur in preparation for a subsequent fear reaction [59]. Therefore, the reduced HR in our study during the anticipation phase in mute children with SM could also be explained by freezing. Further experimental research involving a sufficiently large sample of children with SM is needed to disentangle the underlying mechanisms of avoidance and freezing and to identify possible subgroups. Since only some of the children with SM have failed to speak in our study, whether the discussed mechanisms only occur in a (extreme socially anxious) subgroup of children with SM or whether these mechanisms are inherently related to SM, but can only be observed if failure to speak actually occurs, is still questionable. Here, it would be particularly interesting to investigate whether the children with SM who spoke in our verbal stress paradigm show similar mechanisms in the situations in which they fail to speak (e.g., at school), or whether there are different subgroups with specific mechanisms.

### Clinical implications

Our study has several implications for clinical practice and future research. The finding that children with SM may show increased tonic arousal even in familiar surroundings in the absence of social threat might highlight the importance of defocused communication and anxiety reduction during therapy even in supposedly non-anxious situations. The indication that children with SM show restricted autonomic flexibility, especially in non-verbal social situations, could be an indicator for a generalized pathological fear response beyond verbal situations. In this respect, exposure, which is a central element of CBT in SM [60], should not only be performed in speech-demanding situations to address the failure to speak but should also be explicitly generalized to non-verbal social situations in order to counteract the pathologically-altered fear reaction. Previous research suggests that non-verbal social situations induce a comparable level of anxiety in children with SM to that in verbal situations [13, 17]. Because different mechanisms may be present in children with SM in verbal and non-verbal situations, a differentiation in therapeutic interventions may be important for both types of situations. In verbal situations, it might be promising to start with therapeutic interventions (e.g., relaxation techniques) in the anticipation of a speech-demanding situation, since a dysfunctional fear response seems to start even before failure to speak occurs. In nonverbal situations, where a restricted autonomic flexibility to stress and thus a delayed adaptation to the social situation seem to be of great relevance, it seems to be promising to use therapeutic techniques that counteract the inhibition of behavior (e.g., motor activation) and create a comfortable atmosphere (e.g. through playful elements). Further research should investigate the underlying mechanisms of SM in different situations and large sample sizes to derive differentiated therapeutic interventions for different potential subgroups.

## Limitations

There are methodological limitations, which confine the conclusions of our study. First, the stress paradigm used in the study is standardized but not validated. However, instructions and procedures were closely aligned with the TSST-C and there was a main effect phase with respect to physiological responses. Therefore, it can be assumed that the paradigms successfully induced social stress. Secondly, the recovery phases in our paradigm were shorter than in previous studies in children with SAD, so that we cannot make any assumption regarding the long-term recovery of the arousal. Thirdly, we have some heterogeneity in our SM group, as we included children with SM and comorbid SAD as well as non-socially anxious children with SM. Although the rate of SAD found in our SM group (80%) is consistent with rates reported in the literature, this heterogeneity did not allow for a clear differentiation between physiological processes in children with both SM and SAD, children with SAD only, and children with SM only and thus disorder-specific conclusions are limited. However, because we performed additional analyses within the SM group and correlational analyses based on symptom scores, we nonetheless also obtained disorder-specific insights based on our results. To fully disentangle disorder-specific mechanisms, future studies should include larger samples with the aforementioned subgroups. Fourthly, it is important to note that we were not able to diagnose autism-spectrum-disorder, speech or language disorders or assess information regarding developmental history based on the clinical interview used in the current study. Furthermore, we also did not capture cognitive abilities of the participating children. Fifthly, given that we chose a rather narrow age range of 8–12 years old due to the influence of age on physiological responses reported in the literature, our results cannot be generalized to other age groups. Therefore, the physiological findings found here and derived explanations for the function of failure to speak cannot be applied to other age groups (e.g. children at the preschool age or adolescents with SM). In order to be able to draw valid conclusions regarding etiological mechanisms of SM, studies with younger children and a longitudinal design would be of great relevance. Sixthly, although there was no experimenter in the room during the measurement at rest, the children may still have experienced participation in the study itself as a socially evaluative setting. However, because the groups did not differ in the subjective level of anxiety during the measurement at rest, it can be assumed that a possibly induced stress level was reduced to a minimum. Seventhly, it is important to emphasize that although we had sufficiently large statistical power for the main analyses, the further analyses of children with SM who spoke and who did not speak were based on only very small sample sizes. Future studies should more closely examine the mechanisms identified in the present study using larger sample sizes.

## Conclusion

In conclusion, this is the first study that examined psychophysiological responses in children with SM differentiated for verbal and nonverbal social stress situations and a non-threatening resting measurement in familiar surroundings. We have addressed various methodological limitations of previous psychophysiological studies with children with SM by carefully controlling for confounding variables such as age, gender, and speech production; used a standardized albeit not validated stress paradigm; considered possible baseline differences; and looked at a detailed course of physiological arousal. We identified increased tonic arousal during a non-threatening resting measurement and restricted autonomic flexibility during nonverbal social situations in children with SM, indicating a pathologically altered physiological response in affected children. Furthermore, we showed that a subgroup of children with SM who did not speak during the verbal stress task showed a reduced physiological response already in anticipation of the speech demand. These results combined may suggest that failure to speak acts as an avoidance mechanism that can inherently function only in verbal situations and counteracts a dysfunctional fear response, whereas in nonverbal situations a proper compensation is absent.

## Supplementary Information

**Additional file 1: Table S1.** Correlation analysis between age, gender, symptom scores of SM, SAD, RIBI and physiological reactivity and recovery during verbal paradigm. **Table S2.** Correlation analysis between age, gender, symptom scores of SM, SAD, RIBI and physiological reactivity and recovery during nonverbal paradigm. **Table S3.** Descriptive statistics of physiological variables. **Table S4.** Comorbidities of clinical groups. **Table S5.** Comparison of the paradigm of the current study and the TSST-C regarding different aspects.

## Data Availability

Not applicable.
