# Supplementary material for: Psychophysiological mechanisms underlying the failure to speak: a comparison between children with selective mutism and social anxiety disorder on autonomic arousal
Source: Child Adolesc Psychiatry Ment Health. 2021 Dec 28;15:81. doi: 10.1186/s13034-021-00430-1 (PMC8715622; doi:10.1186/s13034-021-00430-1)
Supplement: Supplementary file 1 — Additional file 1: Table S1. Correlation analysis between age, gender, symptom scores of SM, SAD, RIBI and physiological reactivity and recovery during verbal paradigm. Table S2. Correlation analysis between age, gender, symptom scores of SM, SAD, RIBI and physiological reactivity and recovery during nonverbal paradigm. Table S3. Descriptive statistics of physiological variables. Table S4. Comorbidities of clinical groups. Table S5. Comparison of the paradigm of the current study and the TSST-C regarding different aspects. [file 13034_2021_430_MOESM1_ESM.docx]

**Table S1**: Correlation analysis between age, gender, symptom scores of SM, SAD, RIBI and physiological reactivity and recovery during verbal paradigm

|  | HR_Reac | | HR_Recov | | SCL_Reac | | SCL_Recov | | RSA_Reac | | RSA_Recov | | |
| --- | --- | --- | --- | --- | --- | --- | --- | --- | --- | --- | --- | --- | --- |
|  | r | p | r | p | r | p | r | p | r | p | r | p |  |
| Age | .151 | .142 | .167 | .103 | .034 | .754 | .028 | .798 | -.061 | .566 | -.032 | .762 |  |
| Gender | -.152 | .140 | -.073 | .481 | .086 | .427 | -.074 | .493 | .097 | .362 | .037 | .732 |  |
| FSSM-SS | **-.236** | **.023** | -.176 | .091 | .006 | .985 | -.102 | .346 | .093 | .389 | .041 | .707 |  |
| SPAI-C | -.103 | .316 | -.153 | .137 | -.053 | .627 | -.076 | .479 | .059 | .579 | .053 | .616 |  |
| RIBI | -.029 | .777 | .052 | .616 | -.023 | .833 | **-.245** | **.022** | -.039 | .712 | -.065 | .542 |  |

*Note*: Reac = Reactivity (Difference of HR, SCL, RSA between Performance and Baseline), Recov = Recovery (Difference of HR, SCL, RSA between Performance and Recovery), FSSM-SS = Frankfurt Scale of Selective Mutism – Severity Scale, SPAI-C = Social Phobia and Anxiety Inventory for Children, RIBI = Retrospective Infant Behavioral Inhibition Scale

**Table S2**: Correlation analysis between age, gender, symptom scores of SM, SAD, RIBI and physiological reactivity and recovery during nonverbal paradigm

|  | HR_Reac | | HR_Recov | | SCL_Reac | | SCL_Recov | | RSA_Reac | | RSA_Recov | | |
| --- | --- | --- | --- | --- | --- | --- | --- | --- | --- | --- | --- | --- | --- |
|  | r | p | r | p | r | p | r | p | r | p | r | p |  |
| Age | -.011 | .913 | .024 | .820 | -.083 | .450 | .011 | .923 | .102 | .341 | .110 | .302 |  |
| Gender | .082 | .429 | .065 | .533 | .065 | .553 | -.009 | .931 | .091 | .931 | .159 | .135 |  |
| FSSM-SS | -.054 | .624 | -.018 | .871 | -.065 | .551 | .039 | .723 | -.123 | .258 | -.009 | .932 |  |
| SPAI-C | -.169 | .101 | -.112 | .280 | -.033 | .766 | -.077 | .482 | -.121 | .255 | -.136 | .200 |  |
| RIBI | .013 | .897 | .013 | .897 | -.149 | .170 | -.010 | .926 | .090 | .399 | .013 | .902 |  |

*Note*: Reac = Reactivity (Difference of HR, SCL, RSA between Performance and Baseline), Recov = Recovery (Difference of HR, SCL, RSA between Performance and Recovery), FSSM-SS = Frankfurt Scale of Selective Mutism – Severity Scale, SPAI-C = Social Phobia and Anxiety Inventory for Children, RIBI = Retrospective Infant Behavioral Inhibition Scale

**Table S3**: Descriptive statistics of physiological variables

| Variable / Phase | *M* (*SD*) |
| --- | --- |
| **HR** |  |
| BL_verb_ | 88.32 (11.09) |
| Ant_verb_ | 89.59 (10.03) |
| Perf_verb_ | 92.05 (9.68) |
| Rec_verb_ | 88.06 (9.86) |
| **SCL** |  |
| BL_verb_ | 10.42 (6.43) |
| Ant_verb_ | 11.16 (6.48) |
| Perf_verb_ | 11.45 (6.68) |
| Rec_verb_ | 10.14 (6.25) |
| **RSA** |  |
| BL_verb_ | 7.75 (1.61) |
| Ant_verb_ | 7.81 (1.41) |
| Perf_verb_ | 7.39 (1.59) |
| Rec_verb_ | 7.75 (1.40) |

| Variable / Phase | *M* (*SD*) |
| --- | --- |
| **HR** |  |
| BL_nonverb_ | 87.93 (9.99) |
| Ant_nonverb_ | 88.87 (9.62) |
| Perf_nonverb_ | 92.44 (9.08) |
| Rec_nonverb_ | 88.19 (9.66) |
| **SCL** |  |
| BL_nonverb_ | 10.60 (7.18) |
| Ant_nonverb_ | 11.17 (7.12) |
| Perf_nonverb_ | 10.71 (6.66) |
| Rec_nonverb_ | 10.24 (6.76) |
| **RSA** |  |
| BL_nonverb_ | 7.82 (1.50) |
| Ant_nonverb_ | 7.57 (1.52) |
| Perf_nonverb_ | 7.00 (1.73) |
| Rec_nonverb_ | 7.79 (1.58) |

*Note*: RSA = respiratory sinus arrhythmia, HR = heart rate, SCL = skin conductance level, SM = selective mutism, SAD = social anxiety disorder, TD = typical development, BLverb = baseline phase during verbal stress paradigm, BLnonverb = baseline phase during nonverbal stress paradigm, Antverb: anticipation phase during verbal stress paradigm; Antnonverb: anticipation phase during nonverbal stress paradigm; Perfverb: performance phase during verbal stress paradigm; Perfnonverb: performance phase during nonverbal stress paradigm; Recverb: recovery phase during verbal stress paradigm; Recnonverb: recovery phase during nonverbal stress paradigm

**Table S4**: Comorbidities of clinical groups

| Comorbidities within SM and SAD group | SM | SAD |
| --- | --- | --- |
| Social anxiety disorder | 24 | - |
| Separation anxiety disorder | 7 | 4 |
| Specific phobia | 6 | 2 |
| Generalized anxiety disorder | 1 | 3 |
| Enuresis | 3 | 1 |
| Encopresis | 1 | 0 |
| Oppositional defiant disorder | 3 | 2 |
| Transient tic disorder | 2 | 4 |
| Obsessive compulsive disorder | 0 | 1 |
| Disruptive mood dysregulation disorder | 1 | 1 |
| Insomnia | 1 | 1 |
|  |  |  |

Note: SM = selective mutism; SAD = social anxiety disorder

**Table S5**: Comparison of the paradigm of the current study and the TSST-C regarding differen aspects

|  | **Paradigm of the current study** | **TSST-C** | **Rational behind the adaptation** |
| --- | --- | --- | --- |
| Content of stress tasks | Retelling and completing a story (verbal)  Painting a picture (nonverbal) | Completing a story (verbal)  Count loudly (verbal) | Differentiation between verbal and nonverbal social stress |
| Phases of stress tasks | Baseline, Anticipation, Performance, Recovery | Baseline, Anticipation, Performance, Recovery | No adaptation made |
| Order of stress tasks | Consecutively in randomized order | First story than counting | Given that we aimed at differentiating between verbal and nonverbal content, we had to control for order effects |
| Setting | One room at the families’ home | Two standardized rooms in a laboratory setting | The study took place in the families' homes |
| Social evaluative elements | Video recording Social-evaluative phrases | Video recording Social-evaluative phrases | No adaptation made |
